# Cervical Glycosaminoglycans and Extracellular Matrix Remodeling: New Insights and the Therapeutic Promise of Tafoxiparin

**DOI:** 10.3390/cells14241934

**Published:** 2025-12-05

**Authors:** Wojciech Flis, Mateusz Wartęga, Julia Sowińska, Maciej W. Socha

**Affiliations:** 1 Department of Obstetrics and Gynecology, St. Adalbert’s Hospital, Gdańsk Copernicus Healthcare Entity, Jana Pawła II 50, 80-462 Gdańsk, Poland; wflis@copernicus.gda.pl (W.F.);; 2 Department of Perinatology, Gynecology and Gynecologic Oncology, Collegium Medicum in Bydgoszcz, Nicolaus Copernicus University, Łukasiewicza 1, 85-821 Bydgoszcz, Poland; 3 Department of Pathophysiology, Faculty of Pharmacy, Collegium Medicum in Bydgoszcz, Nicolaus Copernicus University, M. Curie-Skłodowskiej 9, 85-094 Bydgoszcz, Poland

**Keywords:** cervical ripening, extracellular matrix, glycosaminoglycans, hyaluronan, heparan sulfate, tafoxiparin, inflammation, labour induction

## Abstract

**Highlights:**

**What are the main findings?**

- Heparan sulfate and hyaluronan dynamically remodel the cervical extracellular matrix and integrate inflammatory and endocrine signals during cervical ripening.
- Cervical glycosaminoglycans emerge as key regulators of collagen disorganization, tissue hydration, and biomechanical softening of the cervix.

**What are the implications of the main findings?**

- Targeting glycosaminoglycan biology offers a novel, mechanism-based strategy for cervical ripening and labour induction.
- Heparan-sulfate-based analogues such as tafoxiparin may provide effective cervical ripening with minimal anticoagulant activity and a favourable safety profile.

**Abstract:**

Cervical ripening is a multifaceted process involving endocrine, inflammatory, and biomechanical signals that act on the cervical extracellular matrix. While previous reviews have focused on hormonal and immune mechanisms, the specific role of cervical glycosaminoglycans (GAGs)—particularly hyaluronan and heparan sulfate—has received limited dedicated attention. This review addresses that gap by exploring how these GAGs function as integrators of hormonal cues, immune activation, and extracellular matrix remodeling during pregnancy and labour. We conducted a narrative synthesis of experimental, translational, and clinical studies on GAG composition, metabolism, and signaling, with particular attention to tafoxiparin, a heparan-sulfate-based compound with minimal anticoagulant activity. Available evidence suggests that alterations in hyaluronan and heparan sulfate content influence collagen disorganization, tissue hydration, immune cell infiltration, and prostaglandin production—collectively contributing to cervical softening and dilatation. Although tafoxiparin may replicate some actions of endogenous GAGs, current clinical data remain sparse and inconclusive. Thus, targeting cervical GAG biology represents a mechanistic yet still investigational strategy, requiring further studies to determine its therapeutic value.

## 1. Introduction

One of the most frequently performed medical interventions in modern obstetrics is labor induction, undertaken in nearly 20% of pregnancies worldwide. This procedure relies on the iatrogenic initiation of regular uterine contractions, typically with oxytocin, leading to vaginal delivery within 24–48 h [1]. Crucially, successful induction depends on cervical ripening, which must precede the onset of effective contractions [2]. Over the years, the use of numerous cervical ripening agents has been implemented. Mechanical devices, prostaglandins, progesterone antagonists, and other methods are used to stimulate cervical ripening, with varying effectiveness and clinical safety profiles [3]. However, despite extensive clinical experience and numerous trials confirming their efficacy, it has still not been possible to clearly demonstrate the dominance of any specific method of preinduction and induction of labor over others [4].

Cervical ripening is a complex perinatal process driven by enzymatic degradation, a local inflammatory response, and apoptosis [5]. Cervical ripening begins spontaneously (or is artificially induced) during the perinatal period [6]. It involves profound structural reorganization of the cervical extracellular matrix (ECM), which softens the cervical tissue and increases its susceptibility to dilation under contractile forces [7]. This process is orchestrated by multiple interconnected biochemical and molecular pathways whose cooperation is required for proper course of cervical ECM remodelling [8,9]. Those processes are under strict influence of regulatory factors such as: cytokines, metalloproteinases, prostaglandins, reactive oxygen species, transcription factors, etc., [7,10]. Although many pathways have been characterized, the regulatory framework remains incompletely understood [10]. A precise understanding of the regulation and course of cervical ripening appears crucial in the context of developing more effective and safe methods of preinduction and induction of labor.

While several reviews have addressed inflammatory and hormonal signaling in cervical ripening, the specific role of glycosaminoglycans (GAGs) in this process remains underrepresented and poorly synthesized. Emerging evidence suggests that GAGs are not merely passive structural components of the cervical ECM, but may act as active regulators of cervical remodeling. By modulating water content, interacting with signaling molecules, and influencing matrix-degrading enzymes, GAGs contribute to the dynamic remodeling of ECM and may thus represent important mediators of this process [11]. Thus, a focused review on this topic is warranted. To our knowledge, no previous review has comprehensively integrated current knowledge on cervical GAGs biology with the potential therapeutic relevance of heparan sulfate–based compounds in cervical ripening.

Efforts continue to identify novel effective and efficient agents capable of stimulating cervical ripening. It has been postulated that tafoxiparin, through its interactions with components of the ECM, may strongly promote cervical ripening. In addition, tafoxiparin is thought to influence the synchronization of myometrial contractility during the active phase of labour [12]. Nevertheless, the precise mechanism of action of this compound and its impact on the biochemical and molecular pathways underlying cervical remodeling remain poorly defined. Given its structural similarity to the glycosaminoglycan heparan sulfate (HS), tafoxiparin is expected to exert biological effects resembling those through which glycosaminoglycans modulate cervical ripening.

The aim of this review is to summarize the current understanding of glycosaminoglycans (GAGs) in the regulation of cervical ripening, with particular emphasis on the emerging role of tafoxiparin as a potential modulator of these biochemical and molecular pathways.

## 2. Structure and Biology of the Cervical Tissue

The cervix, the lower segment of the uterus derived from the Müllerian ducts, is a highly specialized tissue whose structure and function undergo profound changes across a woman’s reproductive lifespan [13]. The cervical canal, which runs along the cervix, is lined with tall columnar epithelium containing a large number of mucinous glands [14]. During pregnancy, the cervical epithelium undergoes marked secretory adaptation, characterized by an increased number of glands, especially toward term. These glands secrete mucus enriched in glycoproteins and plasma proteins, which provides both a physical barrier to ascending pathogens and an immunological defense that shapes the cervical microenvironment. Unlike the myometrium, which is dominated by contractile smooth muscle, the cervix is composed primarily of connective tissue interwoven with a modest proportion of smooth muscle fibers (~10% of dry weight) [14,15]. These fibers are loosely distributed but display a sphincter-like organization near the internal os, suggesting a role in functional competence during pregnancy [16]. Surrounding this framework is a rich vascular, neural, and lymphatic network that provides both mechanical support and immunological surveillance. The cellular component of the cervix is composed of fibroblasts, mast cells, and mast cells.

At the core of cervical architecture lies the extracellular matrix, dominated by collagen types I and III, with type IV collagen contributing to basement membrane organization [17,18]. Type I and type III collagen fibers are distributed (along with fibroblasts) primarily within smooth muscle fiber bundles [14,17]. Collagen fibers (unlike smooth muscle fibers) are tightly organized in space. The dense, covalently cross-linked collagen fibrils confer mechanical rigidity and resistance to enzymatic degradation—properties critical for maintaining pregnancy [19].

The cervical stroma is enriched in GAGs, synthesized and maintained by resident fibroblasts [20]. Among them, dermatan sulfate, heparan sulfate, chondroitin sulfate, and hyaluronan (HA) dominate the non-pregnant cervix, contributing both to tissue hydration and to the viscoelastic properties of the extracellular matrix (ECM) [21]. Through covalent linkage to protein cores, GAGs give rise to proteoglycans, which act as key organizers of the collagen network [22]. Decorin, biglycan, and chondroitin proteoglycans form the structural backbone of the cervical ECM, stabilizing collagen fibrils and shaping the three-dimensional architecture that underlies cervical tensile strength and enabling resistance to enzymatic degradation [21,23]. Beyond their structural role, proteoglycans exert wide-ranging biological functions. Their anionic side chains interact with collagen fibers, growth factors, and cell surface receptors, generating a complex biochemical microenvironment. These interactions not only enhance the resistance of the ECM to enzymatic degradation, but also regulate cell proliferation, migration, adhesion, and growth factor sensitivity. As such, proteoglycans and their GAG chains represent a dynamic interface between the structural integrity of the cervix and its capacity to remodel in response to hormonal and immunological cues [24,25,26].

Elastic fibers, while representing a smaller fraction of the ECM, add an additional layer of biomechanical control [27]. These fibers are composed of long-chain elastin polymers, cross-linked into microfibrils that establish a highly organized spatial architecture [28,29]. In the non-pregnant cervix, microfibrils display a dense and ordered arrangement [30]. As pregnancy advances, however, both the abundance of elastic fibers and the structural integrity of microfibrils decline, giving rise to a disorganized and dispersed matrix. This remodeling substantially contributes to the progressive softening of cervical tissue [31,32].

During the perinatal period, cervical tissue undergoes profound remodeling, marked by a complete reorganization of the ECM. These changes result in a significant loosening of the previously tightly packed structure of the cervix, accompanied by a rapid increase in the compliance of the vaginal portion of the cervix [5]. Histologically, the process is characterized by a decline in collagen content, enhanced proteolytic degradation of collagen fibers, influx of water, and the emergence of a localized inflammatory milieu driven by infiltrating immune cells. Collectively, these dynamic and interdependent biochemical and molecular events are orchestrated by a diverse network of regulatory signals [8,9].

One of the most important histological events accompanying cervical maturation is the reduction in cervical collagen content. This change is achieved by reducing the expression of collagen assembly genes. During cervical ripening, a significant reduction in type I collagen mRNA expression is observed in the cervical stroma [33,34]. A hallmark of cervical ripening is the enzymatic degradation of collagen fibrils, primarily mediated by matrix metalloproteinases (MMPs) [35]. During the perinatal period, increased expression of MMPs can be observed in cervical tissue [36,37,38]. These enzymes, secreted as zymogens by fibroblasts and infiltrating immune cells, acquire activity in the perinatal period and catalyze the breakdown of the highly ordered collagen covalent bonds network leading to disintegration of the previously tightly packed cervical structure [36,39]. The activity and secretion of MMPs are subject to strict, multifactorial regulation. MMPs expression is enhanced by prostaglandins, nitric oxide (NO), inflammatory cytokines (interleukins), reactive oxygen species (ROS), and growth factors [40,41,42]. In turn, their activity is suppressed by progesterone, retinoic acid and specific tissue inhibitors of metalloproteinases (TIMPs) [40,43].

In summary, the cervix constitutes a highly dynamic connective tissue structure, in which the extracellular matrix confers both rigidity and adaptability. Its capacity for rapid and tightly coordinated remodeling during the peripartum period underscores its central, though still incompletely understood, role in reproductive success. Clinically, the ability to modulate cervical remodeling is of considerable relevance: premature or excessive softening predisposes to cervical insufficiency and preterm birth, whereas inadequate remodeling may result in prolonged or obstructed labor. Despite sustained research efforts, the precise regulatory pathways remain only partly delineated. Emerging evidence indicates that ECM homeostasis, together with the orchestration of a localized inflammatory response, represents the dominant axis governing cervical maturation.

## 3. Inflammation During Cervical Ripening

**Figure 1:**
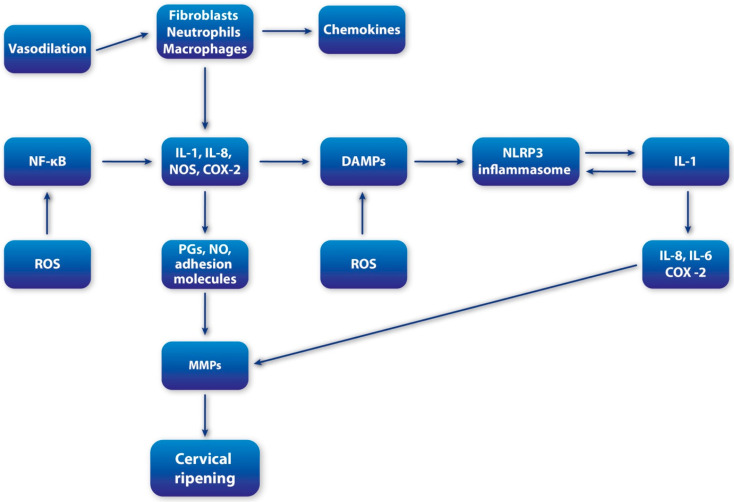
Schematic overview of inflammatory cascade during cervical ripening; MMPs (metalloproteinases); IL-1 (interleukin-1); IL-6 (interleukin-6); IL-8 (interleukin-8); NF-kB (Nuclear Factor-kappaB); COX-2 (cyclooxygenase-2); iNOS (inducible nitric oxide synthase); DAMPS (damage-associated molecular patterns); HS (heparan sulfate); p38MAPK (p38 migoten activated protein kinase); ROS (reactive oxygen species).

Cervical ripening is increasingly recognized as an inflammation-driven process, in which the mediators of immune activation also act as key regulators of ECM remodeling [8]. A large proportion of mediators traditionally associated with the orchestration of inflammatory responses also serve as potent effectors of cervical remodeling, underscoring the central role of immune activation in reproductive tissue adaptation (Figure 1). This perspective has shifted cervical ripening from being viewed as a purely mechanical softening process to being recognized as a highly regulated immunobiological event [44].

At the cellular level, cervical ripening is characterized by the progressive infiltration of the ECM with diverse populations of immune cells. Neutrophils, macrophages, mast cells and fibroblast-like “wandering cells” are particularly abundant in the late stages of pregnancy and act as principal executors of the local inflammatory cascade [45,46,47,48,49]. Their recruitment is facilitated by dynamic changes in the local microenvironment, including upregulation of chemokines, adhesion molecules and vascular permeability, which collectively enhance cellular trafficking to the cervical stroma. Among chemokines, CXCL8 (also known as IL-8) has been highlighted as a powerful chemoattractant for neutrophils, and its increasing expression toward term reflects the escalating inflammatory state [50].

The functional importance of these recruited cells lies in their capacity to secrete a broad repertoire of soluble mediators that directly alter tissue architecture and enhance ongoing inflammation. Macrophages, neutrophils and fibroblasts degranulate to release MMPs, which degrade collagen and other ECM components, thereby weakening cervical tensile strength. Simultaneously, they generate high levels of pro-inflammatory cytokines such as IL-1, IL-6 and IL-8, tumor necrosis factor (TNF) along with prostaglandins, NO and reactive oxygen species (ROS), creating a potent feed-forward loop that accelerates inflammation and tissue remodeling [9,46,51,52,53].

In parallel, non-cellular matrix components actively contribute to this process. Glycosaminoglycans (GAGs), particularly hyaluronan and heparan sulfate, emerge as pivotal regulators [21]. Together, these molecules not only facilitate immune infiltration but also modulate ECM biomechanics, providing a molecular bridge between inflammation and structural softening, which will be discussed in detail in the subsequent sections of this review.

In summary, inflammation is not a secondary phenomenon but a key regulatory mechanism in cervical ripening. It coordinates immune cell infiltration, secretion of cytokines and prostaglandins, and enzymatic remodeling of the extracellular matrix—events that collectively drive the timely and functional softening of cervical tissue to allow for fetal passage [53,54]. Understanding this process requires closer examination of the cellular actors involved (e.g., macrophages, neutrophils, mast cells), the temporal hierarchy of inflammatory mediators (such as IL-6, IL-8, TNF-α, and PGE2), and the reciprocal interactions between inflammatory signals and matrix components [53]. These aspects are explored in the following sections to clarify how inflammation orchestrates the dynamics of cervical remodeling.

### 3.1. Interleukin-1

IL-1 has emerged as a pivotal cytokine in the orchestration of cervical ripening [55]. Experimental studies have demonstrated that intravaginal administration of IL-1 enhances cervical compliance and is accompanied by a marked increase in neutrophil infiltration [56]. These findings highlight the dual capacity of IL-1 to regulate cervical remodeling through both direct and indirect pathways. Direct effects of IL-1 are primarily mediated through modulation of ECM decomposition. IL-1 upregulates MMPs activity while simultaneously suppressing the expression and activity of TIMPs, thereby tipping the balance toward increased collagen degradation and heightened collagenolytic activity within the cervical ECM [57,58,59]. Indirectly, IL-1 exerts a broad spectrum of regulatory influences that further amplify the remodeling process. A central pathway involves its impact on prostaglandin metabolism: IL-1 inhibits prostaglandin dehydrogenase (PGDH), the principal enzyme responsible for prostaglandin inactivation, while simultaneously stimulating the expression of cyclooxygenase-2 (COX-2), the key enzyme in prostaglandin synthesis [60]. The net effect is a robust increase in prostaglandin bioavailability, which accelerates tissue softening and inflammatory signaling. In addition, IL-1 enhances the synthesis of other pro-inflammatory cytokines, including IL-6 and IL-8, thereby amplifying leukocyte recruitment and reinforcing the local inflammatory milieu [61]. Together, these pathways position IL-1 as a central integrator of inflammatory and ECM remodeling signals, bridging cellular immunity with the biochemical processes that drive cervical ripening.

### 3.2. Interleukin-8

IL-8 represents another key cytokine with a central role in cervical remodeling. Its effects extend beyond immune recruitment to include direct modulation of ECM dynamics. Specifically, IL-8 promotes the secretion of MMPs within the cervical stroma, thereby facilitating collagen degradation and structural softening of the cervix [62]. In addition to its matrix-directed effects, IL-8 is a potent amplifier of the inflammatory response. By increasing vascular permeability, IL-8 enhances neutrophil trafficking into the cervical ECM, establishing a robust pro-inflammatory milieu [63]. Importantly, IL-8 does not act in isolation but forms part of an interconnected cytokine network. Evidence suggests that IL-1 not only exerts direct effects on ECM remodeling but also stimulates IL-8 secretion, thereby establishing a positive feedback loop that intensifies local inflammation [64]. The interplay between IL-8 and prostaglandins further illustrates the complexity of this regulatory network. Prostaglandins lower the threshold concentration of IL-8 required to elicit neutrophil chemoattraction, thereby synergistically amplifying leukocyte infiltration [62,65]. Presented interactions underscore the principle that cervical ripening is not driven by isolated mediators but by cooperative signaling pathways that integrate immune activation with ECM remodeling.

### 3.3. Prostaglandins

Prostaglandins represent one of the most powerful mediators of cervical ripening, acting at the intersection of ECM remodeling and inflammatory signaling. Their dual role as physiological regulators and pharmacological agents is well established, as exogenous prostaglandins are widely used to induce labor by promoting cervical softening and dilation [4,52]. At the mechanistic level, their contribution to cervical ripening is multifactorial and extends across both immune and matrix-directed pathways [52]. Within the inflammatory cascade, prostaglandins cooperate with cytokines to enhance vascular permeability, thereby facilitating water influx and the recruitment of inflammatory cells into the cervical stroma [66]. They also promote leukocyte infiltration by increasing the expression of endothelial adhesion molecules, such as intercellular adhesion molecule-1 (ICAM-1), which strengthen interactions between circulating leukocytes and the vascular endothelium [54,67]. In parallel, prostaglandins augment immune cell activity by suppressing secretory leukocyte protease inhibitor (SLPI), a potent inhibitor of neutrophil-derived proteases, thereby intensifying local proteolytic activity [68]. Beyond their immunomodulatory effects, prostaglandins exert direct actions on the cervical ECM. A particularly important interaction occurs with hyaluronan: prostaglandins enhance the expression of neutrophil receptors that bind hyaluronan, thereby potentiating neutrophil degranulation and further reinforcing the inflammatory response [69]. Moreover, prostaglandins directly stimulate the secretion of MMPs accelerating the enzymatic breakdown of collagen and other ECM components [54,70,71,72,73]. This dual capacity to modulate both immune cell function and ECM degradation positions prostaglandins as central orchestrators of cervical remodeling.

### 3.4. Nitric Oxide (NO)

Nitric oxide (NO) has emerged as a critical mediator of cervical ripening, functioning both as a direct regulator of ECM remodeling and as an amplifier of inflammatory signaling [74,75]. The principal sources of NO in the cervix are macrophages and neutrophils, which generate this gaseous mediator through the activity of nitric oxide synthases (NOS). Among the three isoforms of NOS, inducible nitric oxide synthase (iNOS) plays the predominant role in cervical tissue, reflecting the inflammatory nature of the ripening process [76]. Experimental and clinical studies consistently highlight the potency of NO as a cervical ripening agent. Local administration of NO donors effectively accelerates cervical softening, increasing tissue compliance and shortening the time to parturition [77]. Conversely, pharmacological inhibition of NOS suppresses cervical ripening, providing strong evidence for the causal involvement of NO in this process [78]. At the mechanistic level, NO exerts both direct and indirect effects on cervical stroma. Its direct role is mediated through the stimulation of MMPs, which drive the enzymatic breakdown of collagen and other ECM components, leading to progressive reduction in cervical tight structure [79,80,81,82]. Indirectly, NO serves as an upstream regulator of inflammatory mediators. It promotes the secretion of IL-8, thereby enhancing neutrophil recruitment and perpetuating the inflammatory influx into the cervical stroma. In parallel, NO upregulates COX-2 expression in inflammatory cells, which in turn boosts prostaglandin synthesis [83,84,85]. Through this dual regulation of chemokines and prostaglandins, NO integrates into a broader signaling network that synchronizes immune activation with ECM remodeling.

### 3.5. Nuclear Factor-κB (NF-κB)

In addition to the factors described above that directly participate in the development of the local inflammatory reaction (and the course of cervical ripening), it is also worth paying attention to the overarching factors that participate in triggering the inflammatory reaction and in modulating and connecting the cervical ripening pathways.

Nuclear factor-κB (NF-κB) does not represent a single molecule but rather a family of transcription factors composed of distinct subunits, including RelA (p65), RelB, c-Rel, p50 and p52, which can form various homo- and heterodimers with distinct transcriptional activities [86]. Within the cervix, NF-κB has emerged as a pivotal integrator of inflammatory and remodeling pathways, providing a unifying regulatory hub that links upstream signaling events to downstream effector mechanisms of cervical ripening [87]. In the inactive state, NF-κB is sequestered in the cytoplasm through association with inhibitory proteins of the IκB (inhibitor of Kappa-light-chain-enhancer in B cells) family, with the dominance of IκBα isoform, which remains primary regulator of NF-κB activity [88]. The NF-kB activation pathway responds to a variety of stimuli including pattern-recognition receptors (PRRs), cytokines, NO, prostaglandins and TNF receptor (TNFR) superfamily members [89,90]. The IκBα is closely influenced by multi-subunit IκB kinase (IKK) [89,91]. Upon stimulation by diverse signals, the IKK complex becomes activated. IKK phosphorylates IκBα, targeting it for ubiquitination and proteasomal degradation, thereby releasing NF-κB to translocate into the nucleus. Once nuclear, NF-κB binds to κB consensus sequences in DNA and drives the transcription of a broad array of genes essential for cervical remodeling, including iNOS, COX-2, IL-1, IL-6, IL-8, MMPs and NLRP3 inflammasome components [92,93,94]. This broad transcriptional program highlights NF-κB as a master regulator that simultaneously enhances ECM degradation, amplifies leukocyte recruitment and sustains local production of inflammatory mediators. Importantly, accumulating evidence suggests the existence of a self-reinforcing regulatory loop: NF-κB not only induces pro-inflammatory cytokines and enzymes but is itself further activated by these mediators, thereby maintaining its nuclear localization and perpetuating transcriptional activity. This positive feedback may be critical for ensuring the robustness of cervical ripening [95]. Taken together, NF-κB functions as a dominant node in the signaling architecture of cervical ripening. By coordinating the activity of multiple inflammatory mediators and sustaining their synthesis, NF-κB provides the molecular foundation for the ongoing inflammatory response during cervical ripening.

### 3.6. NLRP3 Inflammasome

Another key regulator of the cervical inflammatory response that operates in close synergy with NF-κB is the NLRP3 inflammasome. Inflammasomes are cytoplasmic multiprotein complexes assembled in response to cellular stress signals, including pathogen-associated molecular patterns (PAMPs) and damage-associated molecular patterns (DAMPs) [96]. The typical inflammasome architecture comprises sensor protein (such as NLRP3), the adaptor apoptosis-associated speck-like protein (ASC) containing caspase recruiting domain (ASC) and effector pro-caspase-1 which is responsible for further catalytic functions [97]. The sensor protein acts as pattern-recognition receptor (PRR) which can bind PAMPs and DAMPs. After binding a specific ligand by PRR, caspase-1 is activated, which subsequently cleaves pro-interleukin-1 and pro-interleukin-18 into its mature form—IL-1 and IL-18, which further participates in inflammatory response [96,97,98]. These cytokines, in turn, act as potent amplifiers of inflammation, establishing a feed-forward loop that sustains inflammasome activity, as IL-1 can itself function as a DAMP to further drive NLRP3 activation [98]. Mounting evidence implicates the NLRP3 inflammasome involvement in cervical ripening [99,100]. Elevated levels of IL-1 in the cervical extracellular matrix are consistently observed during late gestation, and inflammasome components—including ASC and caspase-1—have been detected in maternal-fetal compartments such as the fetal membranes, uterine body and cervix at term pregnancy [101,102,103]. These findings strongly suggest that inflammasome assembly and activation are integral to cervical tissue remodeling at term.

The influence of NLRP3 activation on cervical maturation appears multifactorial. According to the research, NLRP3-sufficient (compared to NLRP3-defficient) mice showed greater expression hyaluronan synthase (HAS) genes, suggesting that NLRP3 inflammasome may directly enhance HA synthesis in cervical ECM and participate in cervical ECM remodelling [103]. In parallel, inflammasome-derived IL-1 drives the secretion of other pro-inflammatory cytokines, augments MMPs activity and promotes prostaglandin synthesis while limiting their degradation—thereby coordinating multiple arms of the remodeling process. Whereas, IL-18 exerts complementary effects, notably through the activation of TNF-α and, more critically, through sustained nuclear translocation of NF-κB, which perpetuates the synthesis of NO, cytokines and prostaglandins [103,104]. Together, these mechanisms establish a dynamic interplay between NLRP3 expression and NF-κB activation. This reciprocal regulation creates a robust molecular circuit that ensures the persistence and amplification of the inflammatory milieu necessary for cervical ECM remodeling.

## 4. Structure and Function of Cervical Proteoglycans and Glycosaminoglycans

Cervical proteoglycans and glycosaminoglycans (GAGs) are the predominant constituents of the cervical extracellular matrix (ECM), and many of the biochemical and molecular pathways that govern cervical ripening are closely linked to these components. The following sections will provide a detailed overview of the biology of GAGs and proteoglycans in the context of cervical tissue.

The ECM of the cervix can be broadly divided into collagenous and non-collagenous elements. Collagen types I, III, and IV provide the primary tensile strength of cervical tissue, whereas GAGs and proteoglycans represent the major non-collagenous fraction [105].

GAGs are long, unbranched polysaccharides composed of repeating disaccharide units, typically formed by uronic acid and amino sugar, connected through glycosidic bonds. Variations in disaccharide composition define the major classes of GAGs: hyaluronic acid (HA), heparan sulfate (HS), chondroitin sulfate (CS), keratan sulfate (KS), and dermatan sulfate (DS) [106,107]. With the exception of HA, all GAGs undergo varying degrees of sulfation, which contributes to their functional diversity [108]. GAGs may also bind to a protein core to generate proteoglycans (PGs), which are further classified into intracellular, extracellular, pericellular, and cell surface-linked subgroups, depending on the location of the protein core [108]. Structural heterogeneity among disaccharide subunits confers distinct biological properties to individual GAGs, and each subgroup is represented in the cervical ECM with dynamic expression profiles across gestation [109]. In pregnancy, all known GAGs are detectable in the cervix, but at term, hyaluronan and heparan sulfate predominate, highlighting their potential importance in the regulation of cervical remodeling [21,110].

The glycoprotein family of glycosaminoglycans (GAGs) is highly diverse in both spatial distribution and functional roles. Owing to the structural heterogeneity of GAG chains and proteoglycans, these molecules participate in a broad spectrum of intra- and extracellular processes. While the protein core of proteoglycans primarily determines their localization, the attached GAG chains mediate the majority of their biological functions. Notably, terminal GAG chains engage in interactions with a wide range of ligands, including growth factors, cytokines, chemokines, receptors, adhesion molecules, and pathogen-derived fragments (alarmins)—thereby modulating key signaling pathways and cellular responses [108,111,112,113].

As outlined above, proteoglycans are macromolecules composed of a core protein covalently linked to one or more GAG chains, and they represent a substantial fraction of the cervical ECM [114,115,116]. Beyond providing bulk structural support, cervical proteoglycans critically shape tissue properties through their highly branched, negatively charged GAG chains, which attract water molecules. This hydrating capacity enables the cervix to maintain resilience and withstand the compressive forces generated by the growing fetus during pregnancy [113,114,115,116,117]. Proteoglycans also act as central regulators of ECM architecture, orchestrating stromal organization at a molecular level [109,114,118]. Among them, decorin—a member of the small leucine-rich proteoglycan (SLRP) family—emerges as the dominant proteoglycan in the pregnant cervix [14,114]. Decorin is characterized by a relatively small protein core with a central leucine-rich domain and a single GAG chain (chondroitin or dermatan sulfate). Its specific structure enables tight covalent interactions with collagen fibers. Rather than engaging in enzymatic crosslinking via lysyl oxidase-mediated oxidation of lysine residues, decorin exerts its structural role by non-covalently binding to the D-periodic region of type I collagen fibrils. This steric interaction regulates the lateral growth of collagen fibrils, promoting the formation of thinner, more organized fibrils and contributing to the biomechanical stability of the cervical stroma [114,119,120]. This pivotal role is underscored by experimental studies, which demonstrate that decrease in decorin levels reduced tissue resistance and led to abnormal collagen fiber morphology [121,122,123].

Pregnancy is characterized by dynamic fluctuations in the composition of proteoglycans and GAGs within the cervix. During gestation, decorin and biglycan predominate, together accounting for more than 90% of total cervical proteoglycan content [114,117,123,124,125], whereas hyaluronan, heparan sulfate proteoglycans, and other proteoglycans constitute approximately 10%. At term, however, this distribution undergoes a profound shift, with a 40–70% reduction in stromal decorin accompanied by a marked increase in hyaluronan and heparan sulfate proteoglycans, such as syndecans (mainly syndecan-3) [114,117,124,126,127]. These changes coincide with a reduction in total collagen content and decreased expression of type I collagen mRNA, collectively reflecting the extensive ECM remodeling that underpins cervical ripening. Changes in proteoglycan composition, and the resulting alterations in collagen architecture, disrupt the previously dense and rigid structure of the cervix, driving ECM rearrangement and tissue softening [5]. Such shifts in proteoglycans distribution provide a mechanistic explanation for the marked changes in cervical biomechanics that accompany ripening [128]. Notably, during the perinatal period, HA and HS (together with their proteoglycan derivatives) become the predominant ECM constituents. Taken together, these observations suggest that cervical proteoglycans and their GAG chains are central to ECM remodeling, with proteoglycans providing the structural scaffold and GAG chains mediating the majority of biological functions [109]. Within this framework, HA and HS, along with their proteoglycan partners, emerge as pivotal regulators of cervical ripening pathways.

## 5. Glycosaminoglycans as Modulators of Cervical Inflammation

Unlike other glycosaminoglycans (GAGs), hyaluronan (HA) lacks sulfate groups and does not associate with protein cores. Instead, it is composed solely of repeating disaccharide units of N-acetylglucosamine and glucuronic acid [21,107]. HA is synthesized by the hyaluronan synthase (HAS) enzyme family, which comprises three isoforms—HAS1, HAS2, and HAS3. In contrast to most GAGs, which are synthesized on protein cores within the Golgi apparatus, HA is produced at the plasma membrane by transmembrane HAS enzymes. Their cytoplasmic active sites alternately add disaccharide substrates to an elongating HA chain, which is extruded directly into the ECM as very large, unmodified, non-sulfated polymers [129,130].

In cervical tissue, HAS2 represents the dominant isoform [131]. Whereas HAS1 and HAS3 remain expressed at low levels irrespective of pregnancy, HAS2 expression rises early in gestation and continues to increase until term [132]. Functionally, HAS2 exhibits the highest enzymatic activity and is primarily responsible for the synthesis of high-molecular-weight HA (HMW-HA), which is associated with tissue hydration and structural properties [132]. In contrast, HAS3 and HAS1 generates low-molecular-weight HA (LMW-HA), typically linked to pro-inflammatory signaling [69,132]. HA concentrations in the cervix can be further enhanced following priming with prostaglandin analogues or antiprogestins [133].

During cervical ripening, hyaluronan (HA) becomes one of the predominant glycosaminoglycans (GAGs) in the cervical stroma and exerts a profound influence on tissue remodeling. Owing to its strong hydrophilic properties, HA forms high–molecular weight, space-filling hydrodynamic structures that enhance tissue hydration, promote viscoelasticity, and disrupt the spatial organization of collagen fibers [132]. In addition to these biophysical effects, HA exerts signaling functions through direct interaction with CD44, a widely expressed cell-surface glycoprotein involved in cell–ECM communication [134,135,136,137]. Engagement of CD44 by HA promotes leukocyte recruitment to sites of local inflammation [131,132]. Moreover, HA–CD44 interactions enhance COX-2 expression, thereby stimulating prostaglandin synthesis—an essential driver of the inflammatory cascade and a central regulator of cervical ripening pathways [108,138].

Another important HA receptor is the receptor for HA-mediated motility (RHAMM). Unlike CD44, RHAMM is expressed both in the nucleus and in the ECM, where it modulates inflammatory signaling and cell motility. HA–RHAMM interaction stimulates secretion and activation of MMP-9, leading to disassembly of collagen fiber cross-links [139]. Moreover, extracellular RHAMM can bind HA and, in cooperation with CD44, promote the recruitment and invasion of fibroblasts and neutrophils into sites of inflammation [137,139,140]. Thus, RHAMM not only independently amplifies local inflammatory responses but also synergizes with CD44 to drive cell migration during cervical remodeling.

In addition to its indirect actions, HA exerts direct effects on cervical remodeling. By inhibiting TIMP-1, HA promotes a marked increase in MMPs activity, thereby accelerating the breakdown of covalent cross-links between collagen fibers [141].

Importantly, the biological activity of HA is strongly size-dependent. During late pregnancy, rising HA concentrations are accompanied by increased hyaluronidase activity, which generates low molecular weight HA (LMW-HA) fragments [21,131,142,143,144]. Hyaluronan undergoes enzymatic degradation by hyaluronidases, producing bioactive low molecular weight (LMW) fragments ranging between 20 and 500 kDa, depending on the isoform and enzymatic context [144]. These fragments, owing to their small size, display distinct pro-inflammatory properties and act as potent modulators of cervical ripening [145]. Thus, while high molecular weight HA primarily contributes to ECM viscoelasticity and leukocyte recruitment, LMW-HA fragments amplify pro-inflammatory signaling, collectively driving cervical remodeling [21,146,147,148,149].

According to experimental evidence, LMW-HA fragments exert potent immunomodulatory functions within the cervix. They enhance vascular permeability and directly activate macrophages—previously recruited by HA and other chemoattractants—stimulating secretion of TNFα, IL-1, IL-6 and MMPs, which contribute to ECM remodeling [144,146,147,148,150]. Mechanistically, LMW-HA can engage NF-κB signaling by increasing IKK activity, leading to IκBα phosphorylation and subsequent nuclear translocation of NF-κB, thereby upregulating transcription of pro-inflammatory mediators [108,151,152]. In addition to direct effects, LMW-HA influences inflammatory pathways indirectly. By enhancing TNFα synthesis, LMW-HA provides a priming signal for NLRP3 inflammasome assembly. Moreover, hyaluronidase-generated LMW-HA fragments can function as DAMPs, binding to PRR domains of the inflammasome to sustain its activity. This amplifies IL-1 release, fueling inflammation and prostaglandin synthesis in the cervical stroma [108,150,152,153,154,155]. Furthermore, evidence suggests that LMW-HA represents the predominant bioactive form of HA during cervical ripening. This concept is supported by clinical and experimental data showing that intracervical administration of hyaluronidase facilitates cervical softening and significantly increases compliance [156,157]. Mechanistically, hyaluronidase-driven enzymatic cleavage of HA generates low-molecular-weight fragments that act as potent mediators, integrating and amplifying diverse biochemical pathways underlying cervical remodeling. Collectively, these findings underscore that HA and its low-molecular-weight derivatives are not merely structural components of the cervical ECM but also dynamic regulators of the inflammatory cascades that drive cervical ripening during the perinatal period.

Another key glycosaminoglycan implicated in cervical remodeling is heparan sulfate (HS). HS biosynthesis occurs predominantly in the Golgi apparatus, where repeating disaccharides of N-acetylglucosamine (GlcNAc) and D-glucuronic acid (GlcA) are assembled and subsequently conjugated to a protein core, generating heparan sulfate proteoglycans (HSPGs) [108,111]. Chain elongation produces mature polysaccharide structures that undergo region-specific N-sulfation, forming so-called *S-domains* [106,108,158]. These domains confer remarkable structural and functional diversity, as the high density of negatively charged sulfate groups enables HS to bind and modulate a wide range of ligands—including growth factors, cytokines, MMPs, ECM proteins, enzymes, and even pathogen-derived molecules—thereby positioning HS as a critical regulator of ECM signaling and tissue dynamics [111,159].

Heparan sulfate (HS) is also actively involved in cervical ripening by orchestrating local inflammatory responses. Through its unique structural properties, HS enhances leukocyte recruitment to the cervix. HS chains, covalently attached to proteoglycan cores, can engage L-selectin on leukocytes, thereby stabilizing leukocyte rolling along the endothelial basement membrane [160,161]. In addition, HS binds and presents cytokines secreted by ECM-resident macrophages, including IL-8, CCL5 and CCL4, to cognate receptors on leukocytes [162,163,164]. This interaction promotes integrin activation (for example, ICAM-1), resulting in firm leukocyte adhesion. Notably, chemokines unable to bind HS fail to efficiently recruit leukocytes [165]. Beyond leukocyte adhesion, HS also regulates cytokine stability and availability. By binding cytokines such as IL-6, HS shields them from enzymatic degradation and sustains their bioactivity at the site of inflammation [166]. Together, these findings suggest that HS functions as a molecular organizer of inflammation—binding, presenting, controlling their accessibility to proteases and maintaining chemokine gradients, and ultimately guiding immune cell migration during cervical remodeling.

In addition to its chemotactic role, HS-mediated presentation of IL-8 can amplify local MMPs activity, thereby promoting ECM degradation and cervical remodeling [62]. HS and HSPGs—particularly syndecan-4—also interact with CD44, a key mediator of leukocyte recruitment, and stimulate prostaglandin synthesis through COX-2 induction, as shown in recent studies [112,161].

Intriguingly, HS also appears to modulate inflammatory pathways through an additional mechanism. According to studies, HS may enhance and sustain the activation of p38MAPK (p38 mitogen-activated protein kinases) leading to an exacerbation of the inflammatory response [167,168,169]. MAPKs constitute a family of protein kinases that play central roles in macrophage-mediated inflammatory responses. Within this family, p38 protein are particularly abundant in macrophages [170,171]. Activated MAPKs can promote the degradation of IκBα, thereby relieving inhibition of the NF-κB complex and facilitating its nuclear translocation, which drives the transcription of key pro-inflammatory mediators, including cytokines (e.g., IL-1, IL-6 and IL-8), COX-2 and iNOS [172]. Beyond this canonical NF-κB pathway, p38MAPK also exerts direct regulatory effects on inflammation. Notably, p38MAPK activation enhances COX-2 expression and amplifies the production of pro-inflammatory cytokines (IL-1, IL-6 and IL-8) [169,173]. In parallel, p38MAPK signaling stimulates the synthesis and secretion of MMP-9, a major effector of extracellular matrix remodeling during cervical ripening [173]. Furthermore, by inducing endothelial vascular cell adhesion molecule-1 (VCAM-1), p38MAPK facilitates leukocyte adhesion and transmigration, thereby strengthening the inflammatory cascade [174].

Intriguingly, p38MAPK activation appears to play a pivotal role in the inflammatory events that accompany cervical ripening. Indeed, studies have demonstrated that term pregnancy is characterized by elevated expression of the active form of p38MAPK (and related kinases within the same family) in the cervical stroma [175,176]. These findings suggest a reciprocal relationship between HS and p38MAPK, whereby HS directly activates p38MAPK signaling. Through this mechanism, HS amplifies inflammatory responses within the cervical stroma, ultimately promoting the tissue remodeling necessary for cervical ripening.

As mentioned earlier, HS and HA become one of the dominant GAGs in the cervix during its maturation. Although HS and HA do not form direct molecular complexes, they exert complementary regulatory roles within the cervical ECM, where they coordinate cellular adhesion, migration, signaling and the development of the local inflammatory microenvironment. This interplay is considered crucial for immunoregulation. By binding CD44, HA initiates leukocyte invasion and, via RHAMM, stimulates the activity of MMPs. HS, in turn, enhances local chemokine gradients and protects chemokines from degradation, thereby stabilizing and regulating the dynamics of the inflammatory process. Moreover, HS-facilitated leukocyte invasion increases the availability of hyaluronidase secreted by infiltrating leukocytes, enabling the degradation of HA into low-molecular-weight (LMW) fragments, which exert additional functions essential for inflammation. At the same time, HS directly modulates the course of cervical inflammation through CD44- and p38MAPK-dependent pathways. Taken together, these findings underscore that HS represents one of the key ECM molecules required for the initiation, maintenance, and regulation of the local inflammatory response in cervical tissue during ripening.

Analogous to hyaluronic acid (HA), which undergoes functional modulation upon enzymatic degradation by hyaluronidase, heparan sulfate (HS) is also susceptible to heparinase-mediated cleavage, thereby reshaping its structural landscape and enabling the emergence of distinct biological functions [177]. Heparanase (HPSE), an endo-β-D-glucuronidase, specifically cleaves the β-(1,4)-glycosidic linkage within HS, producing bioactive fragments of 5–10 kDa [178]. The exact number of saccharide units per fragment may vary depending on sulfation density and disaccharide composition [178]. Owing to the pronounced structural homology between HS and heparin, HPSE also targets heparin, underscoring its central role in remodeling the ECM and modulating diverse biological processes [178].

Heparanase is synthesized in the endoplasmic reticulum of inflammatory cells and fibroblasts and secreted into the ECM, where it emerges as a key regulator of tissue remodeling and inflammation. Its enzymatic activity and secretion are orchestrated by key inflammatory mediators, including IL-1, prostaglandins, ROS and TNFs that are abundantly present and actively engaged in shaping the inflammatory milieu during cervical ripening [177,179,180]. Notably, HPSE genes transcription is further amplified by NF-κB, whose activation is markedly elevated within the cervical ECM under inflammatory conditions [181]. Interestingly, scientific evidence indicates that heparanase levels are elevated in the cervical ECM during the peripartum period, suggesting a potential involvement of HPSE in inflammation during cervical remodeling [182].

HPSE may contribute to the development of the local inflammatory response in the cervical tissue through multiple potential mechanisms. First, the enzymatic activity of HPSE generates low-molecular-weight fragments of HS, which can function as DAMPs. These fragments, by engaging pattern recognition receptors (PRRs), promote the activation and assembly of the NLRP3 inflammasome, thereby driving the production of inflammatory mediators [178]. In addition, active HPSE can trigger p38MAPK signaling, leading to enhanced synthesis and secretion of prostaglandins and pro-inflammatory cytokines [183]. HPSE also directly augments the activity of MMP-9, thereby facilitating enzymatic degradation of collagen covalent bonds within the ECM [184]. Importantly, the direct enzymatic action of HPSE degrades HS and its proteoglycan derivatives, thereby reinforcing the ongoing remodeling of the cervical ECM.

**Figure 2:**
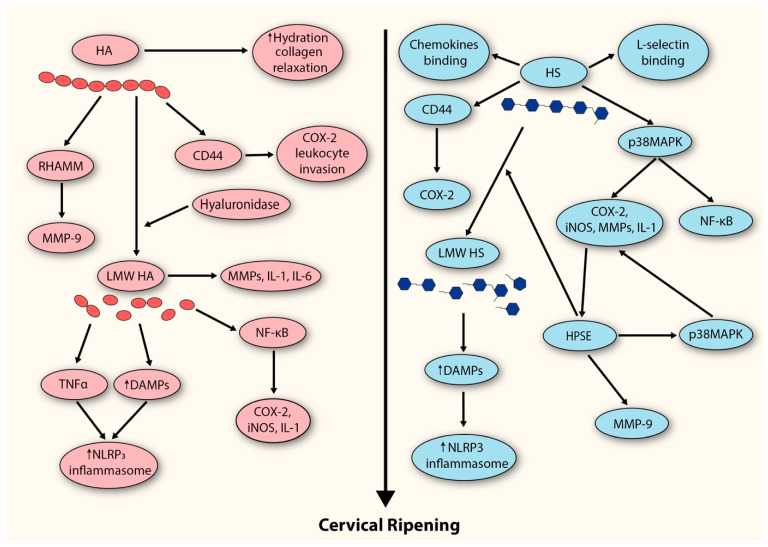
Proposed model depicting the contribution of GAGs to the extracellular matrix remodeling events underlying cervical ripening. HA (hyaluronan); RHAMM (receptor for HA-mediated motility); MMP-9 (metalloproteinase-9); LMW HA (low-molecular-weight hyaluronan); IL-1 (interleukin-1); IL-6 (interleukin-6); NF-kB (Nuclear Factor-kappaB); COX-2 (cyclooxygenase-2); iNOS (inducible nitric oxide synthase); TNF (tumor necrosis factor); DAMPS (damage-associated molecular patterns); HS (heparan sulfate); p38MAPK (p38 migoten activated protein kinase); HPSE (heparanase).

The above findings suggest that both HS and HPSE are actively involved in the process of cervical ripening. However, their contributions do not appear to occur simultaneously. We believe, that during the peripartum period, HS expression (which, together with HA, represents the predominant GAG fraction of the cervix) is upregulated, leading to the development of a local inflammatory response in which these molecules act both directly and indirectly (Figure 2). Subsequently, the influx of inflammatory cells amplifies this response and is accompanied by the release of their granule contents. At this stage, secretion of HPSE (along with hyaluronidase) further amplifies the inflammatory response while simultaneously driving additional ECM degradation through the breakdown of HS and HA.

## 6. Mechanistic Basis of Tafoxiparin Action

Tafoxiparin, a heparin derivative with a slightly lower molecular weight and lacking anticoagulant activity, is structurally similar to both heparan sulfate and its proteoglycans derivatives (mainly syndecan-3) [12,185]. Tafoxiparin is a low-sulfated heparinoid, structurally derived from heparin through partial chemical desulfation [12]. This modification significantly reduces its anticoagulant activity while preserving its biological interactions with heparan sulfate-binding proteins and components of the ECM [185]. Structurally, tafoxyparin retains the linear glycosaminoglycan backbone composed of alternating uronic acid (either iduronic or glucuronic acid) and glucosamine residues—similar to both heparin and endogenous HS [12,185].

Notably, HS appears to represent the predominant glycosaminoglycan within the cervical stroma during cervical ripening, where it plays a pivotal role in ECM remodeling and in modulating inflammatory pathways associated with this process. Owing to its structural similarity to HS (and its HSPGs), tafoxiparin may therefore influence the cervical ECM through mechanisms analogous to those by which HS contributes to cervical ripening.

Based on the biochemistry of glycosaminoglycans and their established role in cervical remodeling, several potential mechanisms can be envisaged. Exogenous administration of tafoxiparin in the perinatal period may increase the effective pool of HS-like molecules within the cervical ECM, thereby accelerating the physiological shift in glycosaminoglycan composition toward HS and hyaluronan [117,186]. This could enhance HS-mediated interactions with L-selectin and cytokines presented by infiltrating macrophages, potentiating leukocyte recruitment and amplifying chemokine gradients. In turn, this would favor secondary leukocyte infiltration, sustained chemotaxis, and intensification of the local inflammatory response. Elevated levels of HS-like molecules may also augment p38MAPK activation, leading to enhanced secretion of IL-1, IL-8, COX-2, and MMP-9, key mediators of cervical tissue remodeling [132,133,134]. Moreover, accumulation of HS-like molecules may provide additional substrates for heparanase, thereby promoting both ECM degradation and HPSE-dependent signaling cascades that coordinate cervical ripening [178]. Finally, it should be noted that the accumulation of HS-like molecules within the cervical ECM may increase their availability as substrates for heparanase, thereby not only enhancing enzymatic degradation of the matrix but also stimulating HPSE secretion. This dual effect is likely to potentiate ECM remodeling while simultaneously activating HPSE-dependent signaling cascades that orchestrate cervical ripening [183].

Importantly tafoxiparin represents a heparin derivative, a characteristic that may endow it with additional functional properties distinct from those arising solely from its structural resemblance to HS. Consistent with experimental observations, administration of low-molecular-weight heparin in the peripartum period has been shown to enhance IL-8 secretion from cervical fibroblasts, thereby amplifying the local inflammatory response [187]. Moreover, heparin has been shown to possess the ability to bind and activate the RHAMM receptor, a resulting in enhanced secretion of MMP-9, the principal enzyme responsible for collagen fiber degradation within the cervical stroma [188]. Finally, heparin may directly stimulate Toll-like receptor 4 (TLR4), a pattern-recognition receptor critically involved in sensing endogenous DAMPs [186]. Its activation promotes the transcription of genes encoding components of the NLRP3 inflammasome, ultimately driving its assembly and activation. As a consequence, this process amplifies the inflammatory response through enhanced synthesis of cytokines and other mediators directly involved in cervical ripening. These pathways suggest that tafoxiparin may integrate both HS-like and heparin-specific mechanisms, thereby converging on multiple nodes of ECM remodeling and inflammation central to cervical ripening.

Taken together, these considerations suggest that tafoxiparin, by virtue of its structural resemblance to both HS and heparin, may represent a unique modulator of cervical ECM remodeling and inflammatory signaling during cervical ripening. However, this hypothesis remains speculative and requires further experimental and clinical validation before any definitive conclusions can be drawn regarding its potential therapeutic applicability.

**Figure 3:**
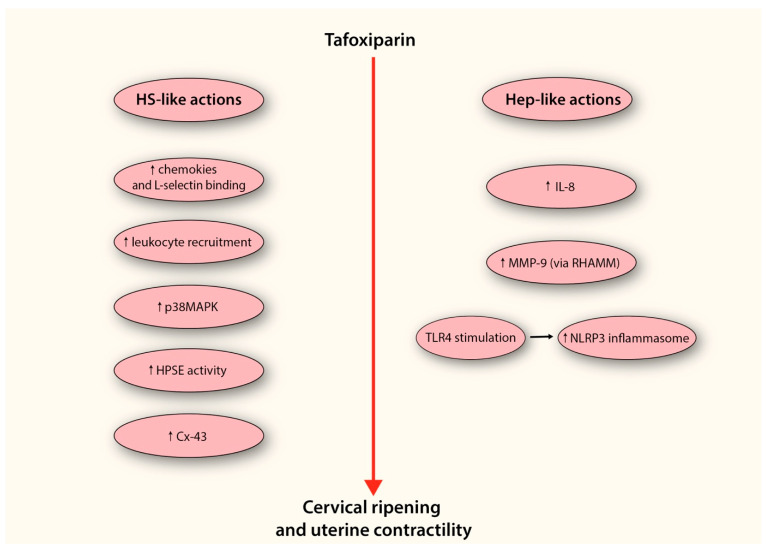
Proposed model depicting the potential mechanism by which tafoxiparin facilitates cervical ripening. The diagram illustrates two parallel mechanistic pathways through which tafoxiparin may exert its effects—one resembling the activity of heparan sulfate (HS-like actions), and the other reflecting its heparin-like properties (Hep-like actions). Both arms converge on enhanced cervical ripening and stimulation of uterine contractility. On the left side, HS-like mechanisms include enhanced chemokine presentation and increased binding to L-selectin, facilitating leukocyte rolling and recruitment into cervical tissue. This cellular influx contributes to the amplification of local inflammation. The accumulation of HS-like structures also leads to the activation of the p38MAPK signaling cascade, which promotes the expression of pro-inflammatory mediators such as IL-1, COX-2, iNOS, and matrix metalloproteinases (MMPs), all of which are critical for ECM remodeling. Moreover, the increased presence of HS-like fragments may provide an additional pool of substrate for heparanase (HPSE), thereby supporting both enzymatic matrix degradation and HPSE-dependent signaling cascades. Through these mechanisms, tafoxiparin may also promote the expression of connexin-43 (Cx-43), a key gap junction protein in myometrial cells, potentially supporting more efficient intercellular communication and synchronization of uterine contractions. On the right side, tafoxiparin’s heparin-like actions involve the stimulation of IL-8 production by cervical fibroblasts, further contributing to leukocyte activation. It also promotes the release of MMP-9 through activation of the RHAMM receptor, enhancing collagen degradation within the cervical stroma. Furthermore, tafoxiparin may activate Toll-like receptor 4 (TLR4), a sensor of endogenous danger signals, thereby promoting transcriptional upregulation of inflammasome components. This, in turn, leads to the assembly and activation of the NLRP3 inflammasome complex, reinforcing the pro-inflammatory environment necessary for effective cervical ripening. Abbreviations: HS-like actions (heparan sulfate-like actions); p38MAPK (p38 mitogen activated protein kinase); HPSE (heparanase); Cx-43 (connexin-43); Hep-like actions (Heparin-like actions); IL-8 (interleukin-8); MMP-9 (metalloproteinase-9); RHAMM (receptor for HA-mediated motility); TLR4 (Toll-like receptor 4).

In summary, tafoxiparin emerges as a promising candidate capable of modulating ECM remodeling and inflammatory signaling during cervical ripening (Figure 3). While these observations highlight its potential as a cervical ripening agent, it is important to emphasize that current evidence remains largely mechanistic and preclinical. Rigorous experimental studies are therefore required to delineate the precise molecular pathways through which tafoxiparin exerts its effects, and well-designed clinical investigations will be indispensable to determine its safety, efficacy, and translational applicability in obstetric practice.

## 7. Discussion

This review aimed to consolidate current insights into the contribution of glycosaminoglycans to cervical ripening, with particular attention to tafoxiparin, a structurally related analogue that is emerging as a promising candidate. Cervical ripening is driven by enzymatic remodeling of the ECM, orchestrated by a diverse array of mediators under the tight control of inflammatory cues. While numerous studies have examined the contribution of individual factors, no single molecular trigger has yet been definitively established as the principal initiator of this process. Our synthesis of the evidence underscores that the major cervical glycosaminoglycans—HS and HA—are actively engaged across nearly all signaling pathways implicated in cervical ripening [134].

The cervix of a pregnant woman differs substantially from that of a non-pregnant one [189]. During pregnancy, profound qualitative changes occur in the composition of the cervical ECM, with a growing predominance of HS and HA [190]. Therefore, it seems reasonable to assume that both HS and HA, together with infiltrating inflammatory cells, represent the main executors of the cervical ripening process. HA, particularly its low-molecular-weight fragments generated by hyaluronidase, contributes not only to tissue hydration and viscoelasticity but also to leukocyte recruitment via CD44 and RHAMM activation [137,140,149]. This interaction enhances COX-2 and MMPs activity, thereby promoting collagen disorganization and local PG synthesis [149]. HS complements these actions by stabilizing chemokine gradients, protecting cytokines from degradation, and directly activating pro-inflammatory signaling pathways, including p38MAPK and NF-κB, which converge on COX-2, MMP-9, cytokines and leukocyte adhesion molecules [152,153]. Importantly, enzymatic cleavage of HA and HS by hyaluronidase and heparanase generates bioactive fragments that act as DAMPs, further amplifying inflammasome activation and sustaining inflammation [97]. Together, these processes position HA and HS not only as structural ECM components but also as active modulators of the inflammatory cascade that underpins cervical ripening.

Beyond their mechanistic role in cervical remodeling, both HA and HS hold promise as potential biomarkers of cervical ripening. Fluctuations in their concentration, molecular size distribution (particularly the generation of low-molecular-weight HA fragments), and enzymatic regulators such as hyaluronidase or heparanase, reflect key biochemical events that precede and accompany labor onset. Therefore, monitoring HA and HS dynamics in cervical tissue or cervical secretions may provide clinically valuable insights into the timing and adequacy of cervical ripening, opening a path toward the development of predictive diagnostic tools and individualized labor management strategies.

A central question that remains is whether alterations in the glycosaminoglycan profile arise as downstream consequences of inflammation, or whether they themselves act as upstream drivers that initiate leukocyte recruitment and prostaglandin production. We believe that hormonal regulation may provide a unifying explanation for this phenomenon, as it plays a pivotal role in orchestrating labor initiation. In the peripartum period, the functional withdrawal of progesterone coincides with a surge in estradiol, which can enhance the transcription of hyaluronan synthase genes, thereby increasing HA levels [191]. Evidence supports this notion, as administration of an estrogen antagonist markedly reduces the expression of HAS2, the predominant enzyme responsible for HA synthesis [21,192,193]. Moreover, studies indicate that estrogen also stimulates the secretion of heparanase (HPSE), leading to HS degradation and the generation of bioactive fragments that further amplify inflammatory signaling [192]. Thus, hormonal regulation may serve as the critical signal driving ECM remodeling toward HS and HA enrichment and turnover, thereby enabling the initiation of their downstream pathways.

Taken together, the evidence positions HS and HA not only as structural scaffolds of the cervical ECM but as active molecular regulators that integrate inflammatory and hormonal signals to drive cervical remodeling [186]. Their dynamic turnover, enzymatic cleavage, and generation of bioactive fragments link ECM biology with immune activation and endocrine control, underscoring their dual role as both effectors and amplifiers of cervical ripening [108]. This interplay highlights the centrality of GAG pathways in orchestrating parturition and sets the stage for exploring targeted therapeutic approaches, including the use of HS analogues such as tafoxiparin [186,188,193]. Although current evidence on the efficacy of tafoxiparin in promoting cervical ripening remains limited, the available data are highly promising and suggest that this HS analogue may exert a significant facilitatory effect on cervical remodeling [12,185].

Previous studies have also examined the efficacy of low-molecular-weight heparin (LMWH), the parent compound of tafoxiparin, suggesting that LMWH may enhance and coordinate myometrial contractility during established labor [187]. Consistently, clinical observations indicate that tafoxiparin shortens the time to delivery, likely through a mechanism analogous to that of heparin [12]. Mechanistically, HS has been shown to increase the expression of gap junctions at the cell surface, and a similar effect within myometrial cells could critically enhance intercellular communication [194]. In particular, connexin-43 (Cx43)–based gap junctions are abundant in the myometrium and indispensable for the proper propagation of contractile signals [195]. Their upregulation would facilitate more efficient transmission of oxytocin-mediated activation pathways, thereby strengthening and synchronizing uterine contractions and ultimately accelerating the course of labor. These findings raise the possibility that tafoxiparin could represent a clinically valuable adjunct or alternative to conventional strategies for cervical ripening and labor induction leading to labor induction optimalization [195].

Despite increasing interest in the role of glycosaminoglycans (GAGs) in cervical ripening, several limitations hinder the development of clinically actionable strategies. First, the vast majority of available studies are preclinical, relying on in vitro models or animal tissues that may not fully recapitulate the complex hormonal and immunological environment of human pregnancy. Second, while evidence supports the involvement of HA and HS in ECM (and its derivatives such as tafoxiparin) remodeling, the temporal dynamics of their expression, cleavage, and functional activity during labor remain poorly characterized in clinical settings.

In terms of clinical translation, the therapeutic potential of tafoxiparin remains underexplored. Current studies are small, heterogeneous, and insufficient to determine efficacy, safety, optimal dosing, or patient selection criteria. Additionally, current evidence is insufficient to support its routine clinical use. Moreover, potential unknown risks associated with its administration—such as immunogenicity or off-target biological effects—have not yet been fully characterized and warrant further investigation.

Furthermore, it remains unclear whether GAG modulation should be pursued as a stand-alone intervention or as an adjunct to established induction agents. Future research should focus on longitudinal, well-powered clinical studies that incorporate standardized sampling and multimodal analysis to characterize the GAG profile across gestation. The integration of biochemical, histological, and functional assessments will be essential to validate GAGs as biomarkers or therapeutic targets. Ultimately, mechanistic studies are needed to determine whether GAG alterations precede or follow the initiation of labor-related inflammation and how hormonal signals integrate into this pathway.

In conclusion, the accumulated evidence underscores that heparan sulfate and hyaluronan are not merely passive structural elements of the cervical ECM but active regulators that integrate inflammatory, endocrine, and mechanical cues to drive cervical remodeling. Their dynamic turnover and bioactive fragments position glycosaminoglycan pathways as central orchestrators of cervical ripening. Within this framework, tafoxiparin emerges as a promising therapeutic candidate, capable of modulating both cervical ripening and myometrial contractility. While current data remain preliminary, they collectively highlight a new paradigm in which targeting glycosaminoglycan biology could offer a novel, mechanism-based strategy for optimizing the induction of labor and management of labor. Future clinical studies will be crucial to validate these insights and to translate them into improved outcomes for both mothers and neonates.

## 8. Conclusions

Cervical ripening represents a dynamic and tightly regulated process that integrates endocrine signals, extracellular matrix remodeling, and inflammation. Within this multifactorial framework, glycosaminoglycans—particularly hyaluronan and heparan sulfate—emerge as active regulators of tissue transformation, linking biochemical, mechanical, and immune pathways. Recent advances highlight the contribution of their low-molecular-weight fragments as amplifiers of inflammation and ECM remodeling. Tafoxiparin, as a structurally tailored HS analogue, offers a promising tool for modulating these pathways, potentially enhancing cervical softening and facilitating labor. Although current evidence remains largely preclinical, these insights point toward glycosaminoglycans as central targets in the development of novel, mechanism-based strategies for labor induction. Further clinical validation will be essential to realize their translational potential and improve maternal–fetal outcomes.

## Data Availability

No new data were created or analyzed in this study.
